# Practice Patterns and Challenges of Glaucoma Specialists in Achieving Optimal Management for Glaucoma in India: Results From a National Survey

**DOI:** 10.7759/cureus.112128

**Published:** 2026-07-06

**Authors:** Parikshit Gogate, Mandar Paranjpe, Ronnie George, Shruti Shah, Pratistha Chippa, Sachin Dharwadkar, Manoj Mathur, Ozukhil Radhakrishnan, Quresh B Maskati, Barun K Nayak

**Affiliations:** 1 Ophthalmology, Dr. D. Y. Patil Medical College, Hospital and Research Centre, Pune, IND; 2 Community Eye Care, Dr. Gogate's Eye Clinic, Pune, IND; 3 Ophthalmology, Paranjpe Eye Clinic and Surgery Center, Pune, IND; 4 Glaucoma Services, Sankara Nethralaya, Chennai, IND; 5 Glaucoma, Dr. Gogate's Eye Clinic, Pune, IND; 6 Ophthalmology, P. D. Hinduja Hospital, Mumbai, IND; 7 Glaucoma, Swarup Eye Centre, Hyderabad, IND; 8 Ophthalmology, Maskati Eye Clinic, Mumbai, IND

**Keywords:** barriers to care, fellowship training, glaucoma practice, glaucoma surgery, gonioscopy, india, perimetry, tonometry, trabeculectomy

## Abstract

Introduction: Glaucoma is one of the leading causes of irreversible blindness in India.

Purpose: This study aimed to explore the current paradigms of glaucoma management among specialists in India and understand patterns of care, barriers to access, and challenges faced by treating physicians.

Design: It is a cross-sectional questionnaire national survey.

Methods: An online questionnaire was distributed to the members of the Glaucoma Society of India, assessing demographic data, practice settings, diagnostic equipment availability, clinical evaluation methods, treatment approaches, postoperative modification, and postoperative complications. Comparisons were made between different practice settings (government, private, non-governmental organizations (NGO)) and between specialists with long-term (>4 months) fellowship training. The chi-squared test was used to determine statistical significance.

Results: Of 159/700 respondents (22.7% response rate), 101 (63.5%) were women. Practice distribution included private practice, 91 (57.2%), NGO/trust hospital, 38 (23.98%), and government settings, 27 (17.02%). Sixty-eight (53.5%) had done a long-term fellowship (>4 months), 16 (12.6%) had done a short-term fellowship (<4 months), and 35 (27.6%) had not done any fellowship. Most specialists had access to Goldmann applanation tonometers, 131 (82.1%), and optical coherence tomography (OCT), 120 (75.5%), but selective laser trabeculoplasty (SLT) availability was limited, 36 (22.5%). Fundus photography (p=0.039) was more frequently performed in private and NGO centers as compared to government hospitals, while Schiotz tonometer (p=0.039) was used more frequently in government facilities. Long-term fellowship-trained specialists performed gonioscopy significantly more often than non-fellowship-trained specialists (65% and 96% vs. 19% and 63.5%; p<0.001). Medical management was preferred by 143 (91.7%), with prostaglandin analogues as first-line treatment by 119 (83.2%). Major patient-related challenges reported included financial constraints, 98 (61.5%), patient knowledge gaps, 98 (61.5%), treatment non-compliance, 96 (60.3%), and late diagnosis due to advanced presentation, 92 (58.3%).

Conclusion: While most Indian glaucoma specialists follow standard practice guidelines, variations exist based on training background and practice setting. Specialized fellowship training significantly improves adherence to best practices.

## Introduction

Glaucoma is the leading cause of irreversible blindness in the world and a significant/main cause of permanent irreversible blindness in India [1-4]. It forms a significant proportion of the workload of ophthalmology/eye departments in general hospitals and private practice in India. While there have been numerous studies on barriers to cataract surgery, surgical techniques of cataract, cause of poor outcomes after cataract surgery, and clinical audits to improve surgical results [5-7], there have been few such studies of glaucoma management [8,9]. There are numerous studies looking into the glaucoma practice patterns in developed countries like Australia, New Zealand, Canada, the United States, the United Kingdom, and Japan [10-19], a few from Nigeria [20,21], and only one so far from India [22].

Glaucoma causes irreversible blindness, and the incurably blind persons are a burden to their family, their community, and the country. Their care involves the use of scarce resources, time, and money that could have been spent on other activities. The visually impaired and blind due to glaucoma are more likely to suffer from anxiety and depression, necessitating psychological help [23]. Their productivity decreases, and many may be out of work, imperiling their financial and social independence. Glaucoma is a leading cause of blindness in the African and East Asian populations [24]. The Rapid Assessment of Avoidable Blindness (RAAB) India studies have shown that glaucoma is a significant cause of blindness in India and visual impairment has increased over the decades [3,4]. The Glaucoma Society of India, the International Society for Geographical and Epidemiological Ophthalmology, and the American Academy of Ophthalmology have issued guidelines for glaucoma management. A study from Pune had shown that late presentation of glaucoma and higher risk of blindness were associated with poverty and lack of knowledge, on the part of both patients and some treating doctors [9]. A survey of glaucoma specialists was done during the annual meeting of the Glaucoma Society of India in 2013 [22]. There are no studies documenting glaucoma management patterns in the public sector, the private sector, non-governmental organizations (NGO), and the corporate sector.

This study aimed to explore the current paradigms of glaucoma management among glaucoma specialists (members of the Glaucoma Society of India) and to understand what the patterns of care are. The secondary objective was to look into the type of medical care and surgical care, barriers to access, equipment, and instrumentation, and challenges faced by treating doctors.

## Materials and methods

Between September 2023 and February 2024, a questionnaire (see Appendices) was delivered to the ophthalmologists who were members of the Glaucoma Society of India. It was sent by email as a Google Form (Google LLC, Mountain View, California, United States) and as a link via SMS and WhatsApp (Menlo Park, California, United States). A reminder was sent by SMS and WhatsApp after one week. 

Permission was obtained from the Institutional Ethics Sub-Committee of Dr. D. Y. Patil Medical College, Hospital and Research Centre (approval number: IESC/28/2021). The Academics and Research Committee of the All India Ophthalmological Society was asked to help popularize data collection and remind the ophthalmologists to participate in the study. There were no financial rewards to participate, the respondents were allowed anonymity, and strict confidentiality of the data was maintained.

The questionnaire was designed to be completed in under 25 minutes, and respondents were allowed to give multiple answers to the questions. Basic demographic data was recorded. The respondents were asked regarding their type of practice (whether government practice, private practice, or working in an NGO) and the duration of glaucoma fellowship they had undertaken (whether long term (>4 months), short term (<4 months), or no fellowship). The practitioners were asked about their daily outpatient department (OPD) load of general and glaucoma patients and monthly cataract and glaucoma surgeries performed.

The equipment availability at the centers was asked about. The equipment asked for included Schiotz tonometer, rebound tonometer, Perkins tonometer, non-contact air puff tonometer, Goldmann applanation tonometer, fundus camera, fundus/goniolens, direct ophthalmoscope, indirect ophthalmoscope, pachymeter, neodymium-doped yttrium aluminum garnet (Nd:YAG) laser, selective laser trabeculoplasty (SLT), optical coherence tomography (OCT), Heidelberg retinal tomography (HRT), Octopus perimeter, Humphrey perimeter, and virtual reality (VR) headset perimeter. The method of clinical evaluation was also quizzed. This included optic disc evaluation, gonioscopy, pachymetry, fundus photo, visual fields, and OCT. Routine gonioscopy and the concept of target pressure were specifically asked about.

Inquired about were the preferred first-line medical management, i.e., prostaglandin analogues, beta blockers, alpha agonists, carbonic anhydrase inhibitors, rho kinase inhibitors, or fixed-dose combinations, and the glaucoma practice performed at the center, namely, SLT, Nd:YAG laser peripheral iridectomy (PI), membranectomy, trabeculectomy, modified trabeculectomy (with mitomycin C and releasable sutures), trabeculotomy+trabeculectomy, goniotomy, cyclodestructive procedures such as transscleral cyclophotocoagulation (TSCPC), cyclocryotherapy, or minimally invasive glaucoma surgery (MIGS).

Postoperative modifications were also inquired about. These included releasable suture removal, laser suture lysis, postoperative subconjunctival injections of 5-fluorouracil, bleb needling, bleb revision, or resurgery. Postoperative complications were also asked about. These included postoperative shallow anterior chamber with hypotony, early bleb failure/congested bleb, thin cystic bleb, snuff-out phenomenon, and blebitis/endophthalmitis.

The overall response was recorded. We compared the data on clinical evaluation and management between long-term and non-long-term fellowship-trained specialists. We also compared the clinical practice patterns and equipment availability between different setups. The chi-squared test was used to determine statistical significance.

## Results

Of the 159 responses, 101 (63.5%) were women. Their ages ranged from 27 to 70 years. Sixty-eight (53.5%) had done a long-term fellowship (>4 months), 16 (12.6%) had done a short-term fellowship (<4 months), and 35 (27.6%) had not done any fellowship. In our survey, 27 (17.02%) of the respondents were in a government practice, 91 (57.2%) in private practice, and 38 (23.98%) in NGO/trust hospital practice.

Table 1 shows the availability and use of commonly found OPD diagnostic equipment for glaucoma management. Fundus lenses and applanation tonometry were available and used by the vast majority. There were non-responders, so the totals are not 100%.

**Table 1:** The equipment availability and usage for glaucoma diagnosis There were non-responders, so the totals are not 100%. Nd:YAG: neodymium-doped yttrium aluminum garnet; SLT: selective laser trabeculoplasty; OCT: optical coherence tomography; RNFL: retinal nerve fiber layer; HRT: Heidelberg retinal tomography; VR: virtual reality

Equipment	Available and frequently used	Available but infrequently used	Unavailable
Schiotz tonometer	50 (33.1%)	26 (17.2%)	41 (27.2%)
Rebound tonometer	47 (31.1%)	6 (4%)	47 (31.1%)
Perkins tonometer	69 (45.7%)	8 (5.3%)	29 (19.2%)
Non-contact air puff tonometer	103 (67.5%)	3 (2%)	22 (13.9%)
Goldmann applanation tonometer	124 (82.1%)	3 (2%)	10 (6.6%)
Fundus camera	110 (72.8%)	5 (3.3%)	17 (11.3%)
Fundus lens	130 (86.1%)	3 (2%)	9 (6%)
Direct ophthalmoscope	107 (70.9%)	19 (12.6%)	9 (6%)
Indirect ophthalmoscope	123 (81.5%)	8 (5.3%)	9 (6%)
Pachymetry	112 (74.2%)	4 (2.6%)	11 (7.3%)
Nd:YAG laser	113 (74.8%)	7 (4.6%)	13 (8.6%)
SLT	34 (22.5%)	4 (2.6%)	54 (35.8%)
OCT RNFL	114 (75.5%)	6 (4%)	12 (7.9%)
HRT	10 (6.6%)	7 (4.6%)	62 (41.1%)
Octopus perimetry	23 (15.2%)	2 (1.3%)	61 (40.4%)
Humphrey perimetry	95 (62.9%)	7 (4.6%)	20 (13.2%)
VR headset perimeter	21 (13.9%)	6 (4%)	53 (35.1%)

Table 2 shows the use of various modalities for glaucoma management. Optic disc evaluation was done almost always, while gonioscopy was always done by three-fourths of the participants, and visual fields were done by 80% of the participants. 

**Table 2:** Overall pattern of clinical practice for glaucoma investigations: always performed, occasionally performed, and rarely performed OCT: optical coherence tomography; RNFL: retinal nerve fiber layer

	Always	Occasionally	Rarely
Disc evaluation	141 (93.4%)	1 (0.7%)	-
Gonioscopy	112 (74.2%)	19 (12.6%)	7 (4.6%)
Pachymetry	103 (68.2%)	26 (17.2%)	6 (4%)
Fundus photo	72 (47.7%)	52 (34.4%)	13 (8.6%)
Visual fields	120 (79.5%)	16 (10.6%)	3 (2%)
OCT RNFL	101 (66.9%)	34 (22.5%)	3 (2%)

Table 3 shows the differences among various types of practice with respect to the use of modalities for the diagnosis of glaucoma.

**Table 3:** Difference in clinical practice patterns among various types of practices *: statistically significant p-value OCT: optical coherence tomography; RNFL: retinal nerve fiber layer; NGO: non-governmental organization

	Government hospital	Private practice	NGO/trust hospital	Chi-squared value	P-value
Disc evaluation					
Always	25 (96.2%)	81 (100%)	34 (100%)	4.45	0.108
Occasionally	1 (3.85%)	0	0		
Rarely	0	0	0		
Gonioscopy					
Always	18 (69.2%)	63 (81.8%)	30 (88.2%)	3.58	0.466
Occasionally	6 (23.1%)	10 (13%)	3 (8.8%)		
Rarely	2 (7.7%)	4 (5.2%)	1 (2.9%)		
Pachymetry					
Always	13 (56.5%)	64 (82.1%)	25 (75.8%)	8.16	0.086
Occasionally	9 (39.1%)	10 (12.8%)	7 (21.2%)		
Rarely	1 (4.3%)	4 (5.1%)	1 (3%)		
Fundus photo					
Always	6 (25%)	49 (61.3%)	17 (51.5%)	10.67	0.039*
Occasionally	15 (62.5%)	24 (30%)	13 (39.4%)		
Rarely	3 (12.5%)	7 (8.8%)	3 (9.1%)		
Visual fields					
Always	19 (76%)	70 (88.6%)	30 (88.2%)	3.04	0.551
Occasionally	5 (20%)	8 (10.1%)	3 (8.8%)		
Rarely	1 (4%)	1 (1.3%)	1 (2.9%)		
OCT RNFL					
Always	17 (70.8%)	60 (75%)	24 (70.6%)	1.77	0.779
Occasionally	6 (25%)	18 (22.5%)	10 (29.4%)		
Rarely	1 (4.2%)	2 (2.5%)	0		

Table 4 compares the equipment availability and usage among different types of practices (government, private, and NGO).

**Table 4:** Equipment availability and usage among different types of practices (government, private, and NGO) *: statistically significant p-value Nd:YAG: neodymium-doped yttrium aluminum garnet; SLT: selective laser trabeculoplasty; OCT: optical coherence tomography; HRT: Heidelberg retinal tomography; VR: virtual reality; NGO: non-governmental organization

		Government hospital	Private practice	NGO/ trust hospital	Chi-square value	p-value
Schiotz tonometer	Available	14 (58.3%)	26 (38.8%)	10 (38.5%)	10.09	0.039*
	Available but infrequently used	5 (20.8%)	11 (16.4%)	10 (38.5%)		
	Unavailable	5 (20.8%)	30 (44.8%)	6 (23.1%)		
Rebound tonometer	Available	7 (41.2%)	25 (43.9%)	15 (57.7%)	5.42	0.247
	Available but infrequently used	0	3 (5.3%)	3 (11.5%)		
	Unavailable	10 (58.8%)	29 (50.9%)	8 (30.8%)		
Perkins tonometer	Available	13 (72.2%)	32 (55.2%)	24 (82.8%)	9.95	0.041*
	Available but infrequently used	1 (5.6%)	4 (6.9%)	3 (10.3%)		
	Unavailable	4 (22.2%)	22 (37.9%)	2 (6.9%)		
Goldmann applanation tonometer	Available	23 (92%)	72 (91.1%)	29 (90.6%)	4.02	0.403
	Available but infrequently used	0	1 (1.3%)	2 (6.3%)		
	Unavailable	2 (8%)	6 (7.6%)	1 (3.1%)		
Fundus camera	Available	19 (82.6%)	63 (82.9%)	28 (84.8%)	2.07	0.772
	Available but infrequently used	0	3 (3.9%)	2 (6.1%)		
	Unavailable	4 (17.4%)	10 (13.2%)	3 (9.1%)		
Fundus lens	Available	24 (92.3%)	75 (92.6%)	31 (91.2%)	3.78	0.436
	Available but infrequently used	0	1 (1.2%)	2 (5.9%)		
	Unavailable	2 (7.7%)	5 (6.2%)	1 (2.9%)		
Direct ophthalmoscope	Available	24 (92.3%)	60 (78.9%)	23 (71.9%)	7.71	0.103
	Available but infrequently used	0	11 (14.5%)	8 (25%)		
	Unavailable	2 (7.7%)	5 (6.6%)	1 (3.1%)		
Indirect ophthalmoscope	Available	23 (92%)	73 (91.3%)	27 (79.4%)	7.45	0.114
	Available but infrequently used	0	3 (3.8%)	5 (14.7%)		
	Unavailable	2 (8%)	4 (5%)	2 (5.9%)		
Pachymetry	Available	16 (84.2%)	66 (89.2%)	30 (90.9%)	4.06	0.397
	Available but infrequently used	0	2 (2.7%)	2 (6.1%)		
	Unavailable	3 (15.8%)	6 (8.1%)	1 (3%)		
Nd:YAG laser	Available	23 (92%)	62 (83.8%)	28 (84.8%)	2.98	0.562
	Available but infrequently used	0	4 (5.4%)	3 (9.1%)		
	Unavailable	2 (8%)	8 (10.8%)	2 (6.1%)		
SLT	Available	8 (50%)	12 (24.5%)	14 (51.9%)	7.59	0.108
	Available but infrequently used	0	3 (6.1%)	1 (3.7%)		
	Unavailable	8 (50%)	34 (69.4%)	12 (44.4%)		
OCT	Available	19 (86.4%)	66 (86.8%)	29 (87.9%)	4.46	0.348
	Available but infrequently used	0	3 (3.9%)	3 (9.1%)		
	Unavailable	3 (13.6%)	7 (9.2%)	1 (3%)		
HRT	Available	3 (21.4%)	4 (8.9%)	3 (15%)	3.06	0.548
	Available but infrequently used	1 (7.1%)	3 (6.7%)	3 (15%)		
	Unavailable	10 (71.4%)	38 (84.4%)	14 (70%)		
Equipment available in glaucoma diagnostics	Available	2 (14.3%)	4 (8.3%)	4 (19%)	1.78	0.776
	Available but infrequently used	1 (7.1%)	4 (8.3%)	2 (9.5%)		
	Unavailable	11 (78.6%)	40 (83.3%)	15 (71.4%)		
Goldmann perimeter	Available	5 (33.3%)	3 (6.4%)	4 (19%)	8.63	0.076
	Available but infrequently used	2 (13.3%)	3 (6.4%)	2 (9.5%)		
	Unavailable	8 (53.3%)	41 (87.2%)	15 (71.4%)		
Octopus perimeter	Available	7 (43.8%)	13 (26%)	3 (15%)	4.50	0.342
	Available but infrequently used	0	1 (2%)	1 (5%)		
	Unavailable	9 (56.3%)	36 (72%)	16 (80%)		
Humphrey perimeter	Available	13 (68.4%)	55 (79.7%)	27 (81.8%)	3.46	0.483
	Available but infrequently used	1 (5.3%)	3 (4.3%)	3 (9.1%)		
	Unavailable	5 (26.3%)	11 (15.9%)	3 (9.1%)		
VR perimeter	Available	2 (14.3%)	14 (30.4%)	5 (25%)	3.64	0.457
	Available but infrequently used	1 (7.1%)	2 (4.3%)	3 (15%)		
	Unavailable	11 (78.6%)	30 (65.2%)	12 (60%)		
Non-contact tonometer	Available	18 (81.8%)	57 (81.4%)	27 (81.8%)	3.08	0.545
	Available but infrequently used	0	1 (1.4%)	2 (6.1%)		
	Unavailable	4 (18.2%)	12 (17.1%)	4 (12.1%)		

Table 5 shows the differences among long-term glaucoma fellowship graduates and others with respect to the usage of various modalities in the diagnosis of glaucoma. Those with long-term glaucoma fellowship do gonioscopy more often.

**Table 5:** Difference in clinical practice patterns between long-term glaucoma fellowship graduates and non-long-term glaucoma fellowship graduates *: statistically significant p-value OCT: optical coherence tomography; HRT: Heidelberg retinal tomography

	Long-term glaucoma fellowship graduates	Non-long-term glaucoma fellowship graduates	Chi-squared value	P-value
Disc evaluation				
Always	75 (100%)	66 (98.5%)	1.12	0.288
Occasionally	0	1 (1.5%)		
Rarely	0	0		
Gonioscopy				
Always	72 (96%)	40 (63.5%)	24.17	<0.001*
Occasionally	3 (4%)	16 (25.4%)		
Rarely	0	7 (11.1%)		
Pachymetry				
Always	59 (80.9%)	44 (71%)	2.12	0.346
Occasionally	12 (16.4%)	14 (22.6%)		
Rarely	2 (2.7%)	4 (6.5%)		
Fundus photo				
Always	38 (51.4%)	34 (54%)	4.06	0.132
Occasionally	32 (43.2%)	20 (31.7%)		
Rarely	4 (5.4%)	9 (14.3%)		
Visual fields				
Always	67 (89.3%)	53 (82.8%)	3.79	0.151
Occasionally	8 (10.7%)	8 (12.5%)		
Rarely	0	3 (4.7%)		
OCT RNFL				
Always	52 (70.3%)	49 (76.6%)	1.59	0.452
Occasionally	21 (28.4%)	13 (20.3%)		
Rarely	1 (1.4%)	2 (3.1%)		

Table 6 shows the differences among long-term glaucoma fellowship graduates and others with respect to surgical management or interventions for glaucoma control.

**Table 6:** Differences in surgical management or intervention between long-term glaucoma fellowship graduates and non-long-term glaucoma fellowship graduates *: statistically significant p-value

		Long-term glaucoma specialists	Non-long-term glaucoma specialists	Chi-squared value	P-value
Gonioscopy before surgery	Always	71 (98.6%)	45 (69.2%)	22.76	<0.001*
	Occasionally	1 (1.4%)	16 (24.6%)		
	Rarely	0	4 (6.2%)		
Target pressure	Always	71 (98.6%)	57 (87.7%)	6.69	0.035
	Occasionally	1 (1.4%)	7 (10.8%)		
	Rarely	0	1 (1.5%)		
Releasable suture removal	Occasionally	10 (18.9%)	12 (35.3%)	3.15	0.207
	Often	37 (69.8%)	18 (52.9%)		
	Rarely	6 (11.3%)	4 (11.8%)		
Laser suture lysis	Occasionally	12 (27.9%)	12 (40%)	8.88	0.012*
	Often	16 (37.2%)	2 (6.7%)		
	Rarely	15 (34.9%)	16 (53.3%)		
Post-op 5-fluorouracil/mitomycin C	Occasionally	19 (42.2%)	15 (55.6%)	5.24	0.073
	Often	16 (35.6%)	3 (11.1%)		
	Rarely	10 (22.2%)	9 (33.3%)		
Bleb needling	Occasionally	26 (54.2%)	20 (60.6%)	2.03	0.362
	Often	4 (8.3%)	5 (15.2%)		
	Rarely	18 (37.5%)	8 (24.2%)		
Bleb revision/resurgery	Occasionally	13 (29.5%)	9 (34.6%)	0.40	0.820
	Often	3 (6.8%)	1 (3.8%)		
	Rarely	28 (63.6%)	16 (61.5%)		
Gonioscopy on patient follow-up	Always	51 (81%)	46 (76.7%)	0.91	0.635
	Occasionally	10 (15.9%)	13 (21.7%)		
	Rarely	2 (3.2%)	1 (1.7%)		

For management, 143 (91.7%) preferred medical management, and prostaglandin analogues were used by a vast majority, 119 (83.2%), as the first drug of choice, followed by beta blockers, 43 (30.1%), fixed-dose combinations, 20 (14%), and carbonic anhydrase inhibitors, 11 (7.7%). To differentiate open-angle and closed-angle glaucoma, 112 (74.2%) reported that they always used gonioscopy, while 19 (12.6%) said they used it occasionally. Nearly all were well-acquainted with the concept of target pressure, and 128 (87.1%) used it all the time, eight (5.6%) used it occasionally, and one (0.7%) used it rarely.

The major patient-related challenges were financial constraints (61.5%), lack of knowledge (61.5%), non-compliance with treatment (60.3%), and irregular follow-up (53.8%). The biggest challenges for healthcare providers were late diagnosis due to delayed presentation of patients (58.3%), lack of training among healthcare personnel (46.8%), lack of proper counselling (39.7%), and non-availability of glaucoma investigation in their healthcare settings (25.6%).

When quizzed about the glaucoma surgeries/procedures done at the center, Nd:YAG PI was the most common, 150 (94.5%), followed by trabeculectomy, 137 (86.6%), modified trabeculectomy, 130 (81.9%), and cyclodestructive procedures, 92 (58.3%). Trabeculotomy+trabeculectomy was performed by 69 (43.3%) and MIGS by 51 (32.3%).

Postoperative hypotony, 49 (30.8%), and early bleb failure, 34 (21.4%), were the two common postoperative complications. The most common postoperative procedures were releasable suture removal, bleb needling, and bleb revision.

## Discussion

This manuscript looked into the glaucoma practice patterns followed by ophthalmologists who had a special interest in glaucoma and were practicing as "glaucoma specialists".

Glaucoma is a chronic disease with varied etiologies and types. Therefore, ophthalmologists need to be trained in its appropriate management and overall patient care. Various previous such surveys among ophthalmologists and glaucoma specialists had revealed varied response rates and diagnostic and treatment protocols among similar cohorts [10-21].

In this online survey among members of the Glaucoma Society of India, the response rate was 22.7%, slightly less than a similar survey in 2013. Choudhari et al. compared the prevailing practice patterns in the diagnosis and management of glaucoma among glaucoma specialists and general ophthalmologists at the Annual Conference of the Glaucoma Society of India in 2013 [22]. The glaucoma specialists were more likely to prefer the Goldmann applanation tonometer, four-mirror gonioscope, Humphrey perimeter, laser PI gonioscopy, and the use of antifibrotic agents during filtering surgery, compared to those without glaucoma training [22]. In our study, the use of gonioscopy before surgery and post-surgery modifications were more common in those who had undergone a long-term glaucoma fellowship.

In this survey, a significantly large percentage of ophthalmologists were following the guidelines laid by the Glaucoma Society of India regarding the use of standardized diagnostic methods like Goldmann applanation tonometer, gonioscopy, pachymetry, optic nerve head evaluation, fundus photography, automated visual field, and optic nerve head imaging.

However, half of the respondent ophthalmologists still used non-Goldman instruments like Schiotz tonometry (more in government setups), non-contact tonometry, and iCare tonometry. One encouraging trend noticed in this survey was that the majority (133/147, 90.5%) used indirect fundus evaluation (90D, 78D) and also a significant number (101/135, 74.8%) used OCT as a diagnostic modality, mirroring similar trends in many developed nations [17-19]. Among the imaging modalities, OCT retinal nerve fiber layer (RNFL) was the most used (vs. glaucoma diagnostics and HRT), as is to be expected, given the recent developments in OCT technology as well as its reliability and availability. Another reason for this may be because, in our survey, only 27 (17.02%) were from government hospitals, while the remaining were from either private practice, NGO hospitals, corporate hospitals, or medical colleges, where equipment availability is better. But even in the long-term fellowship-trained ones, there was a low coverage of imaging-OCT techniques, which can significantly impact the final management in patients with myopia or ocular hypertension.

The difference in clinical practice patterns was that gonioscopy was always performed in NGO (88.2%) and private practice (81.8%) compared to government practice (69.2%). While this is sometimes attributed to the fact that the volume of patients is high in government practice, it is also reported that gonioscopes are not always made available for use [25]. Also, fundus photo was utilized more in private practice (61.3%) compared to NGO (51.5%) and government practice (25%).

Long-term fellowship-trained glaucoma specialists performed gonioscopy more (96%) than those without long-term training (63.5%).

Another feature was that the long-term fellowship-trained specialists often performed bleb modifications in the form of use of releasable sutures (69.8%) and laser suturolysis (37.2%), more so than the non-long-term fellowship-trained specialists (52.9% and 6.7%, respectively). Bleb modifications as above generally help in the long-term success of trabeculectomy [26].

When it came to equipment availability, Humphrey perimetry availability among practices seems uniform; however, use of VR-based perimetry headsets seems to be on the rise in private practice (30.4%) compared to government practice (14.3%) and NGO practice (25%). This could be due to the relatively economical cost and smaller size of VR-based perimeters as compared to cupola perimeters. This should be viewed with caution as there is not much data available from the VR-based headset perimetry and they have a limited range of contrasts available compared to conventional perimeters.

Rhee et al. used the American Society of Cataract and Refractive Surgery (ASCRS) database to study the primary practice patterns for the initial management of primary open-angle glaucoma, with a response rate of 1.3% (252 of 19,246 (1.3%) surveys were returned). Topical medications were the first line of management for 73.6%, followed by laser treatment in 26.4% [17]. This was similar to the findings of our study. Those who practiced in the United States, who had recently completed residency, who had a greater volume of MIGS, and whose glaucoma patient base was greater than 25% were more likely to select laser as compared to drops initially.

Olivieri et al. surveyed glaucoma practice patterns in the Veterans Health Administration in the United States with a response rate of 45% (39/86) [16]. Almost half (49%) believed that laser trabeculoplasty was an option for initial glaucoma therapy. One-third believed there were delays in glaucoma care. Non-compliance with medical treatment was the commonly reported indication for laser trabeculoplasty (95%), tube shunt procedures (65%), microinvasive glaucoma procedures (59%), and trabeculectomy (48.7%) [16]. Non-compliance with treatment was a common barrier reported by ophthalmologists in our study as well.

Coleman et al. in the RiGOR study looked into the practice patterns in open-angle glaucoma in the United States [15]. They reported that in patients who were posted for an intervention or change in medical management in 2016, 36% had two or more therapies over the past year. The most frequent additional medications were prostaglandin analogues (60%), SLT (87%) among those with laser, and trabeculectomy (57%) among those with incisional surgeries.

Skuta et al. compared the academic versus non-academic practices in the United States using the American Academy of Ophthalmology's IRIS registry from 2016 to 2019 [13]. Academy practices had a higher frequency of gonioscopy use, pachymetry, and visual field testing for their new patient visits as compared to non-academic ones. Academic practices had a 2.5-fold higher use of tube shunt procedures, 2.5 times higher use of procedures, and nearly six times more cyclophotocoagulation. Hence, cyclodestructive procedures were left to academic practices perhaps because only they had the equipment.

As far as management is concerned, medical management still remains the first-line choice, given the easy availability and cost of treatment. Prostaglandin analogues (with a 30% reduction in intraocular pressure (IOP)) have been reported as the first line of treatment. A majority of participants were aware of target IOP, a concept important in the optimal care of glaucoma patients. This is in line with reports from the United States [12,15].

Among the surgical procedures, laser PI was the most common, which is due to the higher incidence of primary angle closure (PAC) spectrum of disease in the Indian population vs. the Caucasian population. However, the need for laser PI in every patient of primary angle closure suspect (PACS) is debatable, given the low risk of PACS developing PAC after five years [27]. Almost one-third of the respondents performed some form of MIGS procedure.

Trabeculectomy with or without mitomycin C still remains the preferred incisional glaucoma surgery, a trend dissimilar to the Western world, where drainage devices have started increasing [12,18].

There was reasonable access to cyclodestructive procedures (58%) reported being used as an option for treatment. Cyclodestructive procedures are used for end-stage glaucoma with no or limited visual potential which is not uncommon in the Indian setting, as glaucoma blindness due to poverty and lack of knowledge still persists [8].

Among the postoperative complications, hypotony and bleb failure were most common.

Among the challenges in managing glaucoma patients, financial constraints, lack of knowledge, and non-compliance scored equally. This trend is confirmation of a similar study done in Pune [8]. Patients who are poor, from the lower socioeconomic strata, with less education, and living far away from the hospital were more likely to present with advanced glaucoma and hence more likely to lose vision [8,23].

About 58.3% of the doctors reported late presentation for diagnosis as a barrier to glaucoma care, and 46.8% felt lack of training was a problem in glaucoma management. Thus, better training for general ophthalmologists and awareness regarding the disease in the population should be looked into considering the irreversible nature of the disease [25,28]. Olivieri et al. found that barriers to assessing surgery in the Veterans Health Administration in the United States were lack of transportation (69%), scheduling challenges (62%), and delayed referrals (62%) [16]. A study looking into residency training in the first decade of the 21st century found that while diagnostic equipment availability was high in training institutes, surgical training for glaucoma management was inadequate [28]. Glaucoma surgeries were rarely taught during residency training.

This study has some limitations. Reporting bias is known in survey-based studies. The sample size was not sufficient to reflect the trend in managing glaucoma among a cohort of glaucoma specialists. We could not determine whether the responses of the non-participants differed from those of the participants. Moreover, a multiple-choice format, as used in this study, limits the number of responses to only those offered. Also, exercises like these are prone to recall bias, and as responses were self-reported, there may be a bias to appear more desirable or appropriate. The response rate for this study was less than half the Glaucoma Society of India membership.

However, this study does highlight changing trends among glaucoma specialists and general ophthalmologists in the management of glaucoma in the last decade [22]. This study shows that efforts taken by the member faculty of the Glaucoma Society of India in conducting academic meetings to improve the quality of care in this potentially blinding disease have borne fruit. There was under-utilization of fundus photography. Not all respondents were using applanation tonometry and visual field test which is a sine qua non in glaucoma management. There was a significant difference between long-term fellowship-trained and short-term fellowship-trained doctors, but even in the long-term fellowship-trained ones, there was a low coverage of imaging-OCT techniques which can significantly impact the final management in patients with myopia or ocular hypertension. Underdiagnosis and perhaps overtreatment have been the bane of glaucoma management [29].

## Conclusions

There was a significant difference in long-term fellowship-trained vs. short-term fellowship-trained doctors. Diagnosis of glaucoma is a very labor-intensive process involving the use of multiple investigation modalities having their own importance in the scheme of things. All these tests are not mutually exclusive but contributory to the final diagnosis.

We found that a significant number of practitioners did not have access to or did not use all the pillars to the diagnosis. Underdiagnosis due to limitations in equipment and investigation protocols may be responsible for a significant loss of quality of life years in those patients, a gap that needs to be addressed. Similarly, poor uptake of services by patients needing these compounds the problem of underdiagnosed, undertreated, or mistreated patients, adding a significant burden on the society in terms of the cost of care for terminally blind persons. This can be avoided by better training, proper management, and patient education.

## Appendices

Glaucoma Preferred Practice Patterns

Name of practitioner:

Age:

Gender: Male/Female

Degree (Please select whichever is appropriate): MS/MD   DNB    DOMS

City of practice:

State:

Type of practice (Please tick whichever is most appropriate):

1. Private practice

2. Government hospital: Civil/Rural

3. NGO/trust hospital

4. Corporate hospital

5. Government medical college

6. Private medical college

Nature of practice (Please tick whichever is most appropriate):

1. Urban/city-based

2. Taluka place

3. Peripheral/village level

Area of specialization (Please tick whichever is appropriate):

Glaucoma specialist/Other subspeciality/General ophthalmologist

Whether specialized training was done in glaucoma management: Yes/No

If Yes, nature of fellowship: Long term (>1 year)/Short term (<6 months)

Number of years since the completion of fellowship:

Functioning equipment available at the current place of practice (Please tick whichever is appropriate):

Name of equipment:

Unavailable/Available/Available but infrequently used

Schiotz tonometer:

NCT/air puff tonometer:

Rebound (iCare) tonometer:

Perkins handheld tonometer:

Applanation tonometer (GAT):

Fundus camera:

Fundus lenses (90D/78D):

Direct ophthalmoscope:

Indirect ophthalmoscope:

Gonioscopy lenses:

Pachymetry:

Nd:YAG laser machine:

Abraham iridotomy lens:

SLT:

SLT lens (Ritch, Latina):

OCT RNFL:

Heidelberg retinal tomography (HRT):

Glaucoma diagnostics (GDx):

Visual field analysis:

If available, mention the type:

Manual type (Goldman):

Octopus:

Humphrey visual field analyzer (HFA):

OR:

VR headset bases (Pico, etc.):

Routinely performed evaluations prior to starting therapy in a newly diagnosed glaucoma patient (Please tick whichever is most appropriate):

1. Intraocular pressure: Always/Occasionally/Rarely

2. Clinical disc evaluation: Always/Occasionally/Rarely

3. Gonioscopy: Always/Occasionally/Rarely

4. Pachymetry: Always/Occasionally/Rarely

5. Fundus photo: Always/Occasionally/Rarely

6. Visual fields: Always/Occasionally/Rarely

7. OCT RNFL: Always/Occasionally/Rarely

Glaucoma diagnosis or OPD protocols: 

Approximate number of glaucoma patients seen daily:

Approximate total OPD patients seen daily:

What is your first line of treatment/what glaucoma treatment do you prefer when first diagnosing glaucoma?

1. Beta blockers

2. Prostaglandin analogues

3. Surgery

4. Slit lamp laser trabeculoplasty

Glaucoma procedures you routinely perform at your center (Please tick which is appropriate; can select more than one):

1. Nd:YAG laser PI, membranectomy

2. Selective laser trabeculoplasty (SLT)

3. Trabeculectomy

4. Modified trabeculectomy (with MMC, releasable sutures)

5. Trabeculotomy+trabeculectomy (congenital glaucoma)

6. Goniotomy

7. Cyclodestructive procedures (cyclocryotherapy, TSCPC)

8. Minimally invasive glaucoma surgery (MIGS)

If performing MIGS, please mention the type: 

Glaucoma surgical information:

Average number of cataract surgeries performed in 1 month:

Average number of glaucoma surgeries performed in 1 month:

What are the biggest challenges according to you in glaucoma care and management?

1. Patient-related: 

2. Healthcare provider-related: 

For glaucoma specialists:

Average number of cataract surgeries performed in the last 3 months:

Average number of glaucoma surgeries performed in the last 3 months:

Number of patients counselled for glaucoma surgery in the last 3 months but not operated yet:

Reason for non-compliance according to you (Please tick which is appropriate; can select more than one):

3. Financial constraints

4. Fear of surgery

5. Uncertainty in vision improvement

6. Unawareness/lack of knowledge about glaucoma

7. Medical fitness/systemic factors

8. If any other, please specify:

Follow-up protocols

Do you have a fixed postoperative follow-up schedule? Yes/No

How many visits do you advise in the first 3 months of surgery?

What percentage of patients would you say adhere to follow-ups?

What are the biggest challenges according to you in glaucoma care and management?

Patient-related:

Healthcare provider-related:
